# Environmental and health values, beliefs, norms and compatibility on intention to adopt hydroponic farming among unemployed youth

**DOI:** 10.1038/s41598-024-52064-w

**Published:** 2024-01-18

**Authors:** Jingzu Gao, Abdullah Al Mamun, Qing Yang, Muhammad Khalilur Rahman, Muhammad Mehedi Masud

**Affiliations:** 1 UKM Graduate School of Business, Universiti Kebangsaan Malaysia, 43600 Bangi, Selangor Darul Ehsan Malaysia; 2 Faculty of Entrepreneurship and Business, Universiti Malaysia Kelantan, 16100 Kota Bharu, Malaysia; 3 Angkasa-Umk Research Academy (AURA), Universiti Malaysia Kelantan, 16100 Kota Bharu, Malaysia; 4 Faculty of Business and Economics, University of Malaya, 50603 Kuala Lumpur, Malaysia

**Keywords:** Physiology, Environmental social sciences, Agroecology

## Abstract

The objective of this study was to examine the relationships among environmental and health values, ecological worldview, perception of consequences, the ascription of responsibility, and personal norms in the context of the value-belief-norm (VBN) model and how compatibility influences the intentions and behaviors of Chinese youth regarding the use of hydroponic farming technology. The study employed a survey questionnaire to collect data from the target population. The sample size was determined through a power analysis to ensure sufficient statistical power for the analysis. A total of 727 potential respondents' responses were analyzed using SmartPLS (4.0) to perform structural equation modeling. The results confirmed that environmental, emotional, and health values significantly associated with individuals' ecological worldviews. There was an interconnection between ecological worldview, awareness of consequences, and ascription of responsibility, and all three significantly influenced personal norms. The key determinants of the intentions and behaviors to adopt hydroponic farming technology are personal norms and technology compatibility. Therefore, to promote and motivate the interest and intention to use hydroponics among unemployed youth, government agencies, and related companies should focus on providing technology-related and pro-environmental information and training. This is expected to increase the acceptance and awareness of hydroponics among this group, thus increasing the adoption rate of hydroponics.

## Introduction

The stability and development of agriculture are a constant concern and an important aspect of every country, and the outcome of agricultural development is related to food storage, food security, and food supply in every country^1^. As urban space expands, the global population continues to grow, and the standard of living of the population increases, the demand for food production and food security is increasing, and how to produce more food sustainably and safely is a concern for every country. As the demand for food continues to rise, traditional agricultural practices face challenges such as limited resources such as land and water, environmental pollution, climate change, and pest problems. In response, many countries and regions are exploring and experimenting with emerging agricultural technologies and approaches to promote sustainable and eco-friendly farming practices. Singh et al.^2^ highlighted the growing interest in these innovations to support green agriculture and protect the environment. To solve the employment problem of the unemployed youth and promote new agricultural technologies, China has introduced policies that encourage young people to enter agriculture. Among the emerging agricultural technologies and approaches, hydroponic farming technology is being accepted and adopted by an increasing number of countries and regions with the advantage of saving water and space resources and is gradually becoming a new trend in the agricultural sector^3^.

Hence, the concept of value-belief-norm (VBN) theory may reflect the unemployed youths' intention to adopt hydroponics farming technology as a socio-psychological framework that explains how personal values, beliefs, and norms influence individual behavior. In the context of adopting hydroponic farming, the VBN theory suggests that an individual's values, beliefs, and norms can be linked to their decision to adopt hydroponic farming practices. Values refer to an individual's guiding principles or behavioral standards. For example, people who value sustainability and environmentally friendly practices may be more likely to adopt hydroponic farming, which uses less water and fewer chemicals than traditional farming methods. Therefore, this study investigates the relationship between VBN theory, including environmental values, emotional values, health values, ecological worldview, awareness of consequences, ascription of responsibility, personal norms, hydroponic capability, and the intention of unemployed youths to adopt hydroponic farming technology.

Compared with traditional soil-based farming methods, hydroponic farming methods not only save significant space for cultivation but also enable the sustainable yield and availability of high-quality, healthy^4^, fresh, and chemical residue-free food while reusing water and nutrients, thereby avoiding problems such as soil contamination and pests, as well as negative impacts on the environment^2,3,5^. Currently, hydroponic farming technology is being adopted and promoted in an increasing number of countries and regions and has been gradually developing as the fastest-farming field in agriculture, which in turn can be predicted to have a promising and profitable future in food production^6,7^. In China, the huge population, climate change, and soil erosion and degradation have caused serious challenges to food security and production, which has led the Chinese government authorities to place great emphasis on sustainable agricultural development and the innovation and diffusion of agricultural technologies^8,9^.

Soilless cultivation has become the future trend of agricultural development; therefore, the Chinese government has issued corresponding policies to encourage and support the development and promotion of hydroponic cultivation technology, a new type of soilless agricultural technology^9^. Due to the late development of organic farming, hydroponic agriculture in China is still in the early stages of industrialization. However, driven by green consumer demand, people's awareness of health^10^ and food safety is deepening; they are more willing to choose green and pollution-free food, which, in turn, leads to increased market demand for hydroponic products. This study chooses the Chinese region as the target to understand the current development of hydroponic agriculture in China and the masses' awareness of hydroponic farming technology and to predict the adoption of hydroponic farming technology in the Chinese region and the feasibility of its widespread diffusion.

## Literature review

### Theoretical foundation

In contrast to the self-interest maximization-centered theory of planned behavior (TPB) model, Stern^11^ developed the Value-Belief-Norm (VBN) theory to investigate individuals' behavioral intentions and actual behaviors toward the environment. This model provides a comprehensive explanation of the underlying factors driving altruistic behaviors^12,13^. The VBN theory proposes a causal chain that links an individual's values, belief structures, and norms, supporting and maintaining the environment, with their pro-environmental behaviors^14^. Moreover, this model also accounts for the outcomes of the interaction between humans and the environment.

Individuals' pro-environmental actions are significantly influenced by their values, which are moderated by beliefs and personal norms, such as awareness of consequences (AC) and ascription of responsibility (AR). This view is supported by Li et al.^13^, Stern et al.^14^, López-Mosquera and Sánchez^15^, Choi et al.^16^, Ünal et al.^17^, and Wang et al.^18^. Therefore, a cascading causal relationship exists between individual values, beliefs, personal norms, and pro-environmental behaviors.

Owing to declining traditional industries and the tremendous impact of COVID-19 on the labor market, the number of unemployed youths in China is increasing, and the associated social risks and problems are increasingly surfacing. To accelerate the development of agricultural and rural modernization and increase job opportunities, the government has successively introduced policies to incentivize unemployed youth to engage in agriculture-related entrepreneurship, making agriculture a new blue ocean of innovation and entrepreneurship for much-unemployed youth^19,20^. In China's agricultural development, traditional agriculture is dominated mainly by middle-aged and elderly farmers. It is replaced by new modern agricultural technologies and machinery in the process of iterative renewal of techniques and technologies^9^. Thus, youths with new technologies and multiple skills can apply modern information technology to agricultural production, accelerating the rapid application and widespread diffusion of new technologies in agriculture, facilitating the transformation of traditional to modern agriculture, and opening up sustainable development spaces for green ecological agriculture^20,21^. Therefore, this study aims to explore in depth the perceptions and knowledge of the unemployed youth group on agriculture-related occupations and technologies to examine the acceptance and influential antecedents of agricultural technologies among the Chinese youth group. Then, predict and analyze the possibility of adopting hydroponics for agricultural production among the unemployed youth group and the feasibility of optimizing and developing the agricultural industrial structure.

This study focuses on the unemployed youth group in the Chinese region using the VBN model to study the group's perceptions and intentions toward hydroponic agriculture and farming technology and thus predict the adoption of hydroponic farming technology among the Chinese youth group. Most scholars used the TPB model to study intention and behavior and demonstrated that intention can effectively predict behavior^22,23^. However, to examine in more detail the antecedents that influence individuals' intentions and behaviors toward hydroponic farming technology adoption, this study introduces the VBN model to investigate the influence of factors such as an individual or group's own values, ecological worldview, and personal norms on their intention and behavior toward hydroponic farming technology adoption in water. In addition, this study extends the typical VBN theory by adding technology compatibility to explain individuals' acceptance, intention to use, and behavior toward pro-environmental-related technologies.

### Hypotheses development

#### Values and ecological worldview

Prior studies examining pro-environmental behavior and ecological worldviews have consistently found a strong link between an individual's values and ecological worldview. This connection suggests that individuals with pro-environmental values are more likely to have an ecological worldview that explains the interconnectedness and interactions between humans and the natural environment^11,14,24–27^. Values represent an individual's perceptions and evaluations of the importance of things; as such, they can shape an individual's beliefs and attitudes^12^. By contrast, a worldview is a collection of an individual's concepts, images, values, and beliefs about the world. An ecological worldview refers to beliefs that explain an individual's perceptions and views on the relationship between humans and the natural environment^26,27^.

Individual values can either motivate or inhibit their levels of concern and importance for others and the environment. In turn, these values can influence the ecological worldview regarding the significance of protecting the natural world and subsequently affect pro-environmental behaviors^24–26^. Thus, it is evident that an inseparable relationship exists between individuals' values and ecological worldviews.

Drawing on prior research by Lau et al.^28^, Lee et al.^29^, and Wang et al.^18^, this study categorizes values into three dimensions: environmental, emotional, and health, to examine the impact and interference of these different dimensions on individuals' ecological worldviews. Environmental values refer to an individual's attitudes and beliefs toward environmental protection, influencing their endorsement and support for environmentally responsible behaviors^30^. Emotional values capture an individual's feelings and emotions related to the natural environment, such as a sense of reverence, connection, and responsibility toward environmental protection^31^.

Lastly, health values encompass an individual's attitudes and beliefs toward their own health and well-being, including the importance they place on physical and mental health and their desire for a healthy and sustainable environment^32–34^. These values encompass a sense of responsibility toward the natural world and an understanding of the interdependence between humans and the environment, ultimately shaping an individual's ecological worldview. Han and Hwang^35^ confirmed that values in the VBN theory can influence ecological worldviews, which in turn predict personal norms as a direct antecedent of environmental behavior. Therefore, based on this prior research, the following hypotheses are proposed to explore the relationship between values and ecological worldviews:

*H*_*1–3*_*:* Environmental, emotional, and health values have a positive association with the ecological worldview.

#### Ecological worldview, awareness of consequences, and ascription of responsibility

The ecological worldview in the VBN model is a new environmental paradigm that can primarily explain how the world works and the interdependence and interconnectedness between humans and nature. This is also related to the principle and focused belief that humans and the natural environment live in harmony^11,36–38^. In previous studies, some scholars have verified and identified the relationship between ecological worldview (EW), awareness of consequences (AC), and ascription of responsibility (AR) through VBN models. Denley et al.^37^ demonstrated that tourists' perceptions of environmental issues (i.e., ecological worldview) could lead them to consider the positive/negative consequences of engaging in pro-environmental behaviors, thus enhancing their sense of responsibility to protect the environment and other related behaviors. Some scholars, in their studies on the sustainable and environmental behaviors of tourists and hotel occupants, confirm that tourists' and guests' ecological worldviews can make them aware of the existence and subsequent consequences of environmental problems and then realize their own responsibility to protect the environment^38–41^. Considering that the adoption of hydroponic farming technology is also an environmentally beneficial agricultural method, the ecological worldview of adopters may similarly motivate them to be aware of the consequences of adoption on the environment and to have a higher awareness of the consequences of their behaviors, thereby strengthening their ascription of responsibility for environmental protection. Therefore, the following hypotheses are proposed:

*H*_*4*_*:* The ecological worldview has a positive association with awareness of consequences.

*H*_*5*_*:* Awareness of consequences has a positive association with the ascription of responsibility.

### Beliefs and personal norms

Several previous studies have utilized the value-belief-norm (VBN) theory to illustrate that pro-environmental behavior is impacted by a sequence of causal relationships that involve personal values, beliefs, and personal norms, including the ecological worldview, awareness of consequences, and ascription of responsibility^42^. Additionally, Wu^43^ discovered that ecological worldviews could facilitate the establishment of personal norms, which in turn can influence individuals' pro-environmental behaviors. This concept has been verified in multiple studies on pro-environmental behavior, including citizens' energy-saving behavior^44^, conservation behavior toward biodiversity in rivers^45^, and green consumption behavior^46^. Moreover, the formation of personal norms depends on individuals' perceptions of and sense of responsibility for, their actions and consequences^42^. Some scholars in hospitality and tourism studies have confirmed that the progressive relationship between key factors in the VBN model, such as ecological worldview, awareness of consequences, and ascription of responsibility, can significantly influence the environmental intentions and behaviors of tourists or consumers, moderated by personal norms^38,41,46,47^. Chen^48^ found that AC can positively influence users' attitudes, subjective norms, personal norms, and environment-related behaviors. He and Zhan^49^ also confirmed the effects of AC and AR on consumers' personal norms in a study on the intention and behavior of new energy vehicle use. Thus, in the context of exploring the intentions and adoption behaviors toward hydroponic farming, the following hypotheses are proposed:

*H*_*6–8*_*:* Ecological worldviews, awareness of consequences, and ascription of responsibility have a positive association with personal norms.

#### Personal norms and intention toward hydroponic farming

Previous studies have confirmed that personal norms are one of the most important antecedents of pro-environmental intentions and behaviors at the individual level and that personal norms can often positively influence them, such as green hotel and green restaurant products' sustainable consumption intentions and behaviors^47^, fish conservation-oriented intentions and behaviors^45^, purchase of environmentally friendly products purchase intention^50^ and pro-environmental behavior of Chinese children^43^ According to Riepe et al.^45^, personal norms play a crucial role in shaping individual or group intentions and behaviors toward environmental protection. Personal norms refer to the sense of moral obligation that motivates individuals or groups to support and achieve environmental protection goals. Koklic et al.^51^ also established that personal norms significantly and positively impact consumers' pro-environmental intentions and behaviors. Based on these findings, the present study aims to investigate the relationship between personal norms and adoption intentions of hydroponic farming, leading to the formulation of the following hypothesis:

*H*_*9*_*:* Personal norms have a positive association with intention toward hydroponic farming.

#### Hydroponics compatibility

Considering hydroponics as an emerging agricultural technology, examining and exploration of technological compatibility are essential. Compatibility is used to examine the demand for, perception of, and acceptance of innovative technology by potential adopters^52^. Therefore, this study examines hydroponic compatibility to analyze the acceptance of hydroponic farming technology among unemployed Chinese youth and the extent to which it is consistent with the lifestyle of this group. Thus predicting the impact of hydroponic compatibility on hydroponic farming adoption intentions and behaviors. Technology compatibility has been commonly found in TAM models, and their extensions in past studies, and some scholars have demonstrated the impact of compatibility on user and consumer intentions and behaviors, such as the intention to use e-wallets^53^ using intention and behavior of mobile learning devices^54^, wearable health devices usage intentions^52^ and online shopping intentions^55^. Compatibility can guide or motivate users to change their intention and behavior to use and adopt a product or technology by influencing their sensory perceptions and cognition of the product or innovative technology^55,56^. Thus, considering the possible impact of hydroponic technology compatibility on hydroponic farming technology adoption, this study hypothesizes the following relationship between compatibility, adoption intention, and behavior:

*H*_*10–11*_*:* Hydroponic compatibility has a positive link with the intention and adoption of hydroponic farming.

#### Intention toward hydroponic farming

Pro-environmental behavioral intentions can be viewed as the subjective perceptions of people involved in environmental behaviors, which, in turn, explain the thoughts and tendencies of people involved in pro-environmental behaviors^57,58^. Several studies have utilized the Theory of Planned Behavior (TPB) to establish the interrelatedness between pro-environmental intentions and behaviors, such as fish conservation-oriented intentions and behaviors^45^, consumers' environmental intentions and behaviors^59^, sustainable consumption intentions and behaviors in green hotels^47^, and environmentally friendly product purchase intentions and behaviors^60^. Liu et al.^58^ affirmed that individuals' pro-environmental intentions could directly influence their subsequent behaviors and increase the likelihood of engaging in pro-environmental actions. In the present investigation, intention toward hydroponic farming is posited as a predictive factor to further understand the behavioral motivations of unemployed youth toward adopting hydroponic farming. Thus, we propose the following hypothesis:

*H*_*12*_*:* Intention toward hydroponic farming has a positive association with the adoption of hydroponic farming.

**Figure 1:**
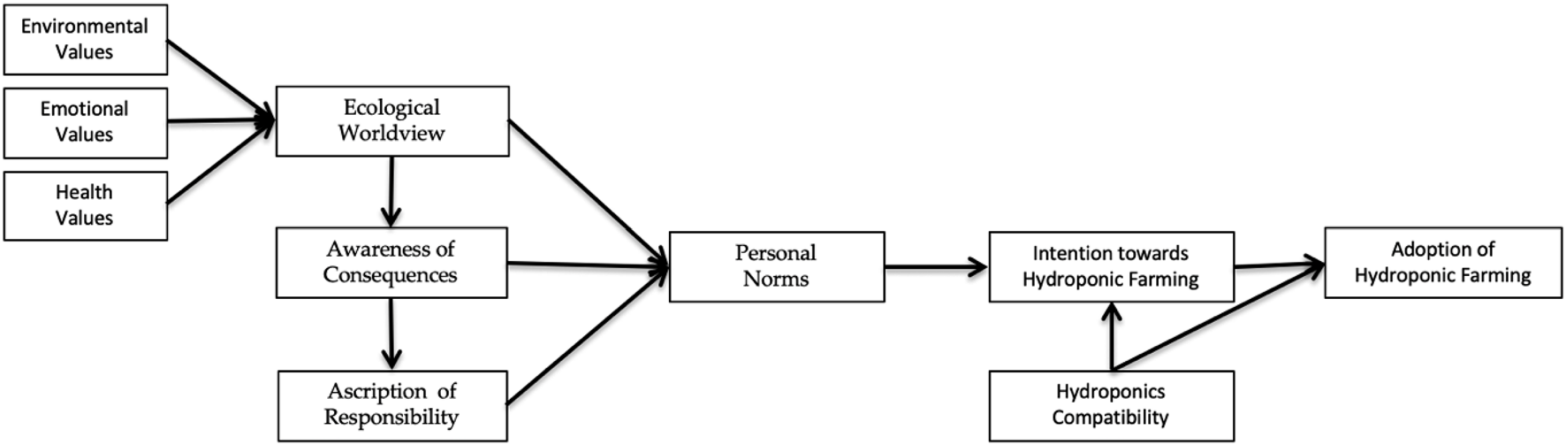
Research framework.

Based on the literature review and theoretical concept, this study proposes the following research framework (see Fig. 1):

## Research methodology

### Data collection method

This quantitative cross-sectional study employed an independent questionnaire as the primary data collection instrument. Questionnaire responses were used to gain a detailed and in-depth understanding of the knowledge and acceptance of hydroponics among unemployed youth in China, as well as the likelihood of future hydroponic farming behavior. The primary sample for this study was either unemployed or under-employed youths in China. The human research ethics committee of Changzhi University have approved this study (Approval Number: CZ-2022–0074). This study has been performed in accordance with the Declaration of Helsinki. Written informed consent for participation was obtained from respondents who participated in the survey. To protect the security of the respondents' personal information and avoid wasting resources on paper questionnaires, all questionnaires were distributed and collected through an online questionnaire platform and software.

The distribution of the questionnaire link was carried out through various channels that were deemed appropriate for the target audience. These channels included: (a) associations representing current students and graduates; (b) popular social media platforms; and (c) online centers for career counseling and platforms for job-seeking. The selection procedure was conducted in a manner that adhered to the principles of voluntary participation and the protection of data confidentiality. Prior to their decision to participate, participants were given a comprehensive comprehension of the content and aims of the questionnaire. Furthermore, it is important to note that all participants were duly informed about their entitlement to access information and the assurance of maintaining the confidentiality of their personal data. A total of 906 questionnaire replies were obtained over the months of March and April 2022. Out of these, 179 questionnaires completed by employed participants were eliminated, leaving a final sample size of 727 valid and usable questionnaire responses. Data was not obtained from individuals who were below the age of 18.

### Instrument

This study designed and improved the questionnaire used by adapting research scales and items used in previous studies, thus ensuring the validity of all items in the questionnaire. The questionnaire for this study was initially written in English as the referenced literature was structured in English. However, to be applicable to Chinese respondents and ensure the accuracy and validity of the questionnaire items, the final Chinese version of the scale (questionnaire) was translated and evaluated by Chinese language experts. The questionnaire comprised three sections: instructions, demographic-related measures, and scale questions. The questionnaire description section was designed to help respondents understand the purpose and content of the survey through a written narrative and then allow them to choose whether they would like to take the survey or not and answer all items according to their own knowledge and experience. The second section consisted of demographic questions related to personal information, such as age and gender, screening items for judgmental sampling (employment status), and items related to hydroponic farming.

The third part of the questionnaire in this study consists of a 7-point Likert scale, ranging from "strongly disagree" to "strongly agree," which was utilized to assess respondents' knowledge and perceptions of factors such as their values, beliefs, and norms, as well as their predictions of hydroponic farming intentions and adoption behaviors. The items included in this section were adapted from previous studies and modified accordingly to fit the research context and the subjects of this study. Each scale contained 4–6 items for the assessment variables. For example, values-related items were adapted from Wang et al.^18^ for EN; Lee et al.^29^ and Sweeney and Soutar^61^ for EM; and Lau et al.^28^ for HV. The EW items were adapted from López-Mosquera and Sánchez ^15^, AC items from Choi et al.^16^, and AR items from Ünal et al.^17^. PN items were adapted from Choi et al.^16^, and Ünal et al.^17^, the items for HC from Al Mamun et al.^62^. The items for intention toward Hydroponic Farming (IT) were adapted from Maichum et al.^63^, and the items for AD were taken from Kim et al.^64^. The complete questionnaire is available in the supporting material.

### Common method bias (CMB)

Kock^65^'s full collinearity test was used to evaluate the potential presence of common method bias. As shown in Table 1, the test yielded variance inflation factor (VIF) values for all items that were below 3.3, which indicates the absence of multicollinearity issues. As recommended by Podsakoff et al.^66^, this suggests that CMB is not a concern for data collected from a single source.

**Table 1:** Kock's full collinearity test.

Variables	EN	EM	HV	EW	AC	AR	PN	IT	HC	AD
VIF	1.384	1.544	1.397	1.505	1.354	1.403	1.444	1.348	1.217	1.557

*EN* environmental values; *EM* emotional values; *HV* health values; *EW* ecological worldview; *AC* awareness of consequences; *AR* ascription of responsibility; *PN* personal norms; *IT* intention toward hydroponic farming; *HC* hydroponics compatibility; *AD* adoption of hydroponic farming.

*Source:* Author's data analysis.

### Multivariate normality

The assessment of multivariate normality is paramount in research endeavors as it facilitates the identification of suitable data analysis techniques. Multivariate normality was evaluated using the Web Power online tool (source: https://webpower.psychstat.org/wiki/tools/index). The outcomes derived from the data processed using the tool revealed that the *p* values associated with all variables were below 0.05. In line with Yang et al.’s.^56^ recommendation, p-values below the 0.05 threshold signify the presence of non-normality, which, in turn, indicates that the data examined in this study exhibits evidence of non-normality.

### Data analysis method

This study effectively tackled the exploratory non-normality issue by employing a variance-based partial least squares structural equation model (PLS-SEM), which was confirmed through rigorous tests. PLS-SEM is commonly adopted for the predictive analysis of complex structures and non-normal data, particularly in the fields of marketing and management^67^. To evaluate the measurement model, this study utilized PLS-SEM, which considered various key aspects including, but not limited to, average variance extraction (AVE), internal consistency reliability, discriminant validity, effect size, predictive correlation, indicator reliability, path coefficient estimation, and convergent validity. Furthermore, to obtain a comprehensive understanding of the participants, this study assessed the predictive correlation of the structural model as advocated by Becker et al.^68^.

## Results

### Demographic characteristics

A total of 906 completed questionnaires were collected for this study, and 727 usable valid questionnaire data were obtained through judgmental sampling. The specific respondent demographics can be found in Table 2. Since the study was conducted with youth, all respondents were 18–35 years old, with the largest number of respondents in the 23–27 age group (50.3%), followed by 28–32-year-olds (34.8%), 18–22-year-olds (6.5%) 18–22-year-olds, and 33–35-year-olds (8.4%). The gender ratio of the respondents in this study was very even, with 52.8% male respondents and slightly fewer female respondents (47.2%). Regarding education level, the majority of respondents (59.6%) had only a diploma degree or less, followed by 33.3% with a bachelor's degree. Of the respondents, 33.2% were from Northeast China, followed by 22.3% from Northern China. In addition, this study was conducted on respondents' access to and consumption of fresh fruits and vegetables during the COVID-19 period in China, where more than half of the respondents (53.6%) felt that consumption was the same as before, and 38.9% felt that it had increased compared to the past. The majority of respondents (51.9%) felt that access to fresh fruits and vegetables was the same as before, but 39.1% thought it had increased compared to the past, and 9.1% thought it had decreased. Moreover, a significant proportion of participants (81%) expressed their belief that they had access to adequate space for hydroponic farming, whereas 72.8% reported receiving prior training in the field of hydroponic farming.

**Table 2:** Respondents’ demographic profile.

	n	%		n	%
Gender			Education level		
Male	384	52.8	Diploma	433	59.6
Female	343	47.2	Bachelor	242	33.3
Total	727	100.0	Postgraduate Degree	50	6.9
			Others	2	0.3
			Total	727	100.0
Age group					
18–22 years	47	6.5			
23–27 years	366	50.3	Access to fresh fruits and vegetables		
28–32 years	253	34.8	Increase	284	39.1
33–35 years	61	8.4	Same	377	51.9
Total	727	100.0	decrease	66	9.1
			Total	727	100.0
Location					
Northeast China	228	31.4	Consumption of fresh fruits and vegetables		
North China	162	22.3	Increase	283	38.9
East China	63	8.7	Same	390	53.6
Central China	60	8.3	decrease	54	7.4
South China	85	11.7	Total	727	100.0
Northwest China	24	3.3			
Southwest China	89	12.2	Access to adequate space for hydroponic farming		
Others	16	2.2	Yes	589	81.0
Total	727	100.0	No	138	19.0
			Total	727	100.0
Employment status					
Unemployed	727	100.0	Training on hydroponic farming		
			Yes	529	72.8
			No	198	27.2
			Total	727	100.0

*Source:* Author's data analysis.

### Validity and reliability

**Figure 2:**
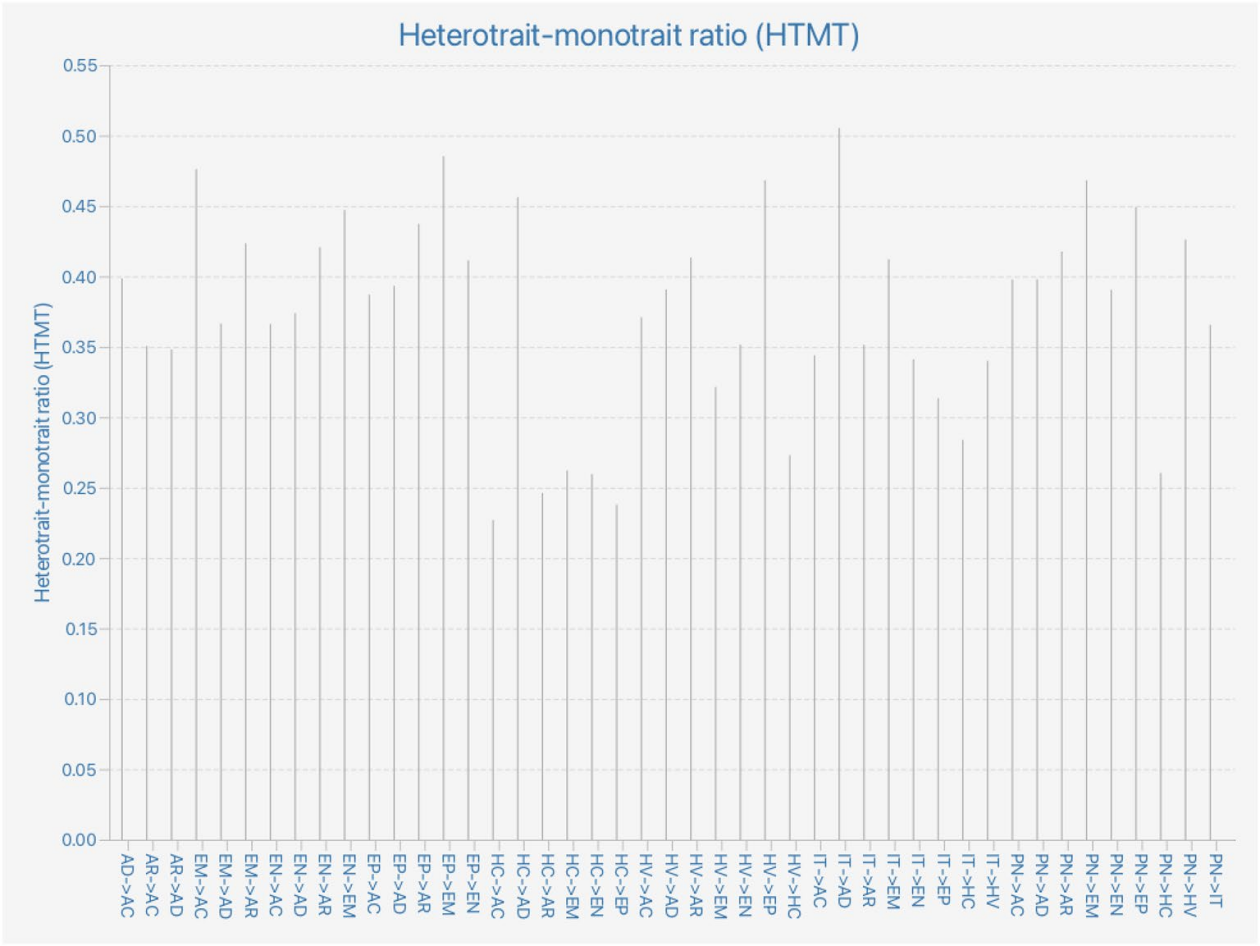
Heterotrait-monotrait ratio (HTMT).

This study took measures to ensure the reliability and validity of the questionnaire, as established by Hair et al.^67^. Specifically, Cronbach's alpha values were computed for all measures and were found to be greater than 0.80, indicating a high level of consistency and reliability of the scale (Table 3). Furthermore, the questionnaire's validity was evaluated by assessing convergent and discriminant validity, with the former measured by the average extracted variance (AVE) and factor loadings, as Hair et al.^67^ recommended. The AVE values recorded in Table 3 exceeded 0.50, indicating acceptable convergent validity^69^. Discriminant validity, however, was assessed using the Fornell-Larcker criterion (see Supporting Material S2. Discriminant Validity) and heterogeneity-monotonicity ratio (HTMT), as proposed by Avkiran^70^. As illustrated in Fig. 2 and Supporting Material S2. Discriminant Validity, the HTMT values for all items were below 0.60 and well below the threshold of 0.85, which suggests that the HTMT between dimensions was within the significant range and that the questionnaire's discriminant validity was satisfactory. The loading and cross-loading values (see Supporting Material S2. Discriminant Validity) indicate that all loading values are greater than 0.5, which is higher than the respective cross-loading values. To obtain a comprehensive understanding of the participants, the measurement model was evaluated using PLS-SEM, which accounted for various aspects, such as AVE, internal consistency reliability, discriminant validity, effect size, predictive correlation, indicator reliability, path coefficient estimation, and convergent validity, as suggested by Becker et al.^68^.

**Table 3:** Reliability and validity.

Variables	Items	Mean	Standard deviation	Cronbach's alpha	rho	Composite reliability	Average variance extracted	Variance inflation factor
EN	4	5.398	1.189	0.887	0.893	0.922	0.747	1.247
EM	5	5.063	1.175	0.883	0.891	0.914	0.680	1.229
HV	4	4.869	1.245	0.869	0.871	0.910	0.717	1.146
EW	5	4.924	1.110	0.868	0.869	0.904	0.654	1.247
AC	6	4.855	1.216	0.854	0.858	0.891	0.578	1.179
AR	5	4.949	1.224	0.871	0.879	0.906	0.660	1.223
PN	5	4.892	1.143	0.858	0.859	0.898	0.638	1.051
IT	5	5.445	0.982	0.802	0.805	0.863	0.559	1.058
HC	5	5.286	1.063	0.840	0.840	0.886	0.610	1.058
AD	4	5.628	1.156	0.890	0.891	0.924	0.752	–

*EN* environmental values; *EM* emotional values; *HV* health values; *EW* ecological worldview; *AC* awareness of consequences; *AR* ascription of responsibility; *PN* personal norms; *IT* intention toward hydroponic farming; *HC* hydroponics compatibility; *AD* adoption of hydroponic farming.

*Source:* Author's data analysis.

### Hypothesis testing

This section evaluates the structural model of this study using the values of the path coefficients R^2^, and f^2^ obtained in Table 4 to test and determine the correlation between the study variables. According to Sarstedt et al.^71^, a higher value of R^2^ indicates a higher explanatory power of the model used in the study; therefore, this study evaluated the predictive power of the structural and analytical model by using the R^2^ values in Table 4. The data in Table 4 show that the R^2^ values recorded for EW (R^2^ = 0.294), AC (R^2^ = 0.113), AR (R^2^ = 0.096), PN (R^2^ = 0.238), IT (R^2^ = 0.122), and AD (R^2^ = 0.276) were greater than zero, indicating that the models used in this study had significant and weak explanatory power. Additionally, this study explained and measured the effect sizes of all predictor variables based on the threshold values of f^2^ studied by Hair et al.^67^, in which the threshold values of f^2^ at 0.005, 0.01, and 0.025 explained the small, medium, and large effects of the measured variables, respectively. The data in Table 4 show that the f^2^ values for all measures are greater than 0.025, which means that the effect sizes of all predictor variables can be inferred to be relatively large.

**Table 4:** Path analysis.

Hypothesis		Beta	Confidence interval	t	*p*	R^2^	f^2^	Decision
H_1_	EN → EW	0.163	(0.095, 0.231)	3.931	0.000		0.030	Accept
H_2_	EM → EW	0.286	(0.217, 0.355)	6.844	0.000	0.294	0.094	Accept
H_3_	HV → EW	0.275	(0.210, 0.346)	6.680	0.000		0.094	Accept
H_4_	EW → AC	0.336	(0.265, 0.408)	7.770	0.000	0.113	0.128	Accept
H_5_	AC → AR	0.309	(0.242, 0.381)	7.327	0.000	0.096	0.106	Accept
H_6_	EW → PN	0.243	(0.178, 0.308)	6.047	0.000	0.238	0.062	Accept
H_7_	AC → PN	0.194	(0.120, 0.270)	4.276	0.000		0.042	Accept
H_8_	AR → PN	0.212	(0.143, 0.280)	5.074	0.000		0.048	Accept
H_9_	PN → IT	0.265	(0.199, 0.331)	6.532	0.000	0.122	0.076	Accept
H_10_	HC → IT	0.175	(0.095, 0.258)	3.528	0.000		0.033	Accept
H_11_	HC → AD	0.312	(0.244, 0.379)	7.602	0.000	0.276	0.127	Accept
H_12_	IT → AD	0.356	(0.289, 0.422)	8.878	0.000		0.165	Accept

*EN* environmental values; *EM* emotional values; *HV* health values; *EW* ecological worldview; *AC* awareness of consequences; *AR* ascription of responsibility; *PN* personal norms; *IT* intention toward hydroponic farming; *HC* hydroponics compatibility; *AD* adoption of hydroponic farming.

Table 4 displays the results of the path coefficient analysis, including the p-values and t-values, based on the study data. Notably, the study found significant positive correlations between the EW and EN (β = 0.163, *p* = 0.000), EM (β = 0.286, *p* = 0.000), and HV (β = 0.275, *p* = 0.000). Furthermore, the study discovered that EW (β = 0.336, *p* = 0.000) was significantly and positively associated with AC and that AC (β = 0.309, *p* = 0.000) positively and significantly influenced AR. Moreover, the study observed that PN could be positively and significantly influenced by EW (β = 0.243, *p* = 0.000), AC (β = 0.194, *p* = 0.000), and AR (β = 0.212, *p* = 0.000). Furthermore, this study found that IT had a positive and significant relationship with PN (β = 0. 265, *p* = 0.000) and HC (β = 0.175, *p* = 0.000), whereas the AD of hydroponic farming was positively and significantly associated with HC (β = 0. 312, *p* = 0.000) and IT (β = 0.356, *p* = 0.000).

## Discussions

This study aimed to investigate the factors that shape the intentions and behaviors of unemployed youth in China regarding the adoption of hydroponic farming technology. By providing empirical evidence and supporting the Value-Belief-Norm (VBN) model, this study endeavors to fully comprehend the interplay of various factors, such as environmental values, emotional values, health values, ecological worldview, awareness of consequences, ascription of responsibility, and personal norms, in driving the adoption intention and behavior toward pro-environmental technologies among the study population. The results of this study support the theoretical and empirical validity of the VBN model in the field of agriculture, emphasizing the importance of factors, such as environmental values, on the intention and behavior of using pro-environmental agricultural technologies. In addition, this study explored individual acceptance and approval of pro-environmental and innovative agricultural technologies by examining technology compatibility. Based on the associations between the factors in the VBN model, all hypothesized relationships proved to be positive and significant; the specific discussion of the correlations is elaborated below.

This study supports the feasibility of the VBN model for studying pro-environmental intentions and behaviors in agriculture. Specifically, values were able to positively and significantly influence the ecological worldview of the unemployed youth group, and all three dimensions of values (environmental, emotional, and health) were effective predictors of the ecological worldview. This is in line with the findings of Xiao et al.^25^ and Gkargkavouzi et al.^26^ that the environmental values, emotional values, and health values of the unemployed youth group are the main antecedents for the formation of their ecological worldview. At the same time, the ecological worldview explains and emphasizes the emotional connection and attachment of humans to the natural world, as well as the recognition of the importance of human health and the health of the environment, which in turn stimulates and motivates individuals to act in ways that promote environmental well-being, such as adopting hydroponic farming techniques instead of traditional soil farming techniques to avoid and reduce environmental problems such as soil pollution and fertilizer pollution.

Furthermore, the interrelationships among the ecological worldview, awareness of consequences, and ascription of responsibility were found to be significant and positively correlated. This is consistent with the results of Megeirhi et al.^38^ and Sharma and Gupta^41^ in their studies on sustainable tourism and green tourism. People or groups with an ecological worldview are more likely to have a higher awareness of the consequences of their actions on the environment and to take responsibility for the consequences of their behaviors, which in turn leads them to recognize the sustainability, protection, and conservation of the importance of natural resources and to place long-term benefits over short-term ones. Consistent with prior empirical research, the present study revealed that the ecological worldview, awareness of consequences, and ascription of responsibility all play a positive and significant role in shaping personal norms, which in turn have an impact on pro-environmental intentions and behaviors^42,44,46^. Therefore, when unemployed youths with a strong ecological worldview possess a clear understanding of the consequences of their actions and feel accountable for their outcomes, they are more likely to form positive personal norms, which subsequently motivates them to engage in environmentally responsible behaviors, such as adopting hydroponic gardening techniques and promoting sustainable environmental practices and solutions.

Similar to the results of past studies on green-product consumption and environmentally oriented intentions and behaviors, personal norms can positively and significantly influence individuals' pro-environmental intentions, which, in turn, effectively and positively predict individuals' pro-environmental behaviors^43,45,47,50^. Therefore, unemployed youths who have ethical and pro-environmental personal norms will intend to protect the environment and engage in pro-environmental behavior under the influence of factors such as their own sense of responsibility and values, that is, choosing hydroponic farming techniques to avoid and reduce environmental pollution problems. In addition, the compatibility of the unemployed youth group with technology can significantly and positively influence the generation of their intention to use hydroponic farming technology and their subsequent adoption behavior. This result is consistent with the findings of Shi et al.^55^, Jeong et al.^52^, and Senali et al.^53^ that when an unemployed youth group accepts and has some knowledge of hydroponic farming technology, they are willing to adopt the technology for profit or to solve environmental problems.

## Implications

### Theoretical implications

Previous research using TPB and knowledge-attitude-behaviour theories examined the usage intention and behavior of China's urban population for hydroponic farming; this highlighted significant theoretical implications for the country's urban and hydroponic farming contexts^62^. In a study conducted by Zhang et al.^72^, the researchers examined the suitability of the TPB and the Value-Belief-Norm (VBN) theory in the context of farmers' adaptation to climate change and its impact on food production within the agricultural sector. The findings of the study indicated that the TPB model demonstrated greater efficacy in predicting behaviors driven by self-interest, specifically climate change adaptation. On the other hand, the VBN theory exhibited superior explanatory power in elucidating altruistic behaviors, particularly mitigation behaviors. Given the existing literature on the viability of the VBN theory in various domains of Chinese agriculture, such as agricultural production^72^, environmental practices^73^, and urban agriculture^62^, this study aims to establish a comprehensive framework that substantiates and affirms the practicality and relevance of the VBN theory in the Chinese agricultural sector. Additionally, this study seeks to demonstrate the efficacy of the VBN theory in elucidating altruistic behaviors.

Meanwhile, since the adoption of a technology depends largely on the user's understanding and acceptance of the technology, this study added a compatibility factor to the VBN model to explore the public's acceptance and understanding of hydroponic cultivation technology. In conclusion, the VBN theory with the added compatibility factor can be used as a reference to promote the use and diffusion of emerging environmentally friendly agricultural technologies.

### Practical implications

From a practical perspective, this study explored and highlighted the antecedents of the intentions and behaviors of the unemployed youth in China to use hydroponics, which in turn provides direction and insights for government departments and agribusinesses to promote and implement hydroponics. The findings of this study also provide a basis for the sustainability and market utility of innovative agricultural technologies and confirm the potential impact and acceptance of hydroponic farming technologies among the youth population. Currently, most regional governments in China strongly support and encourage the development of agriculture-related businesses among youth, creating a favorable environment for the development of China's agricultural industry. Therefore, the discussion and results of this study can provide effective incentives and measures for regional governments, such as providing training and courses related to agricultural technologies to enhance the awareness and acceptance of emerging agricultural technologies among youth groups, as well as the need to make them aware of the profitability and feasibility of such technologies, which in turn can motivate youth groups to adopt agricultural technologies, such as hydroponic farming, for entrepreneurship and other behaviors.

These findings have significant implications for the promotion and development of hydroponic agricultural practices in China. Given the lack of knowledge and high costs associated with this technology, the results of this study can aid the Chinese government in promoting the growth and development of urban hydroponic agriculture. This, in turn, can contribute to the advancement and sustainability of China's agricultural economy and address the critical issues of food security and production in the country. By supporting the adoption of environmentally responsible practices, such as hydroponic gardening, the Chinese government can take a significant step toward achieving sustainable agricultural development and addressing the food security concerns of its growing population.

## Conclusions

This study empirically analyzed the factors influencing the intention and behavior of Chinese unemployed youth groups toward the use of hydroponic farming technology using the VBN model. The results confirmed that environmental values, emotional values, and health values have a significant association with individuals' ecological worldviews. The study's findings revealed a significant and positive relationship among ecological worldview, awareness of consequences, and ascription of responsibility, which are instrumental in shaping personal norms. The results further demonstrated that personal norms, along with technology compatibility, exerted a positive and significant influence on the adoption intentions and behaviors related to hydroponic farming technology. These outcomes substantiate the effectiveness and applicability of the VBN model in agriculture and in gauging individuals' intentions to use agricultural technologies.

### Limitations

While this study provides valuable insights into the factors influencing the adoption of hydroponic farming among unemployed youths in China, it has certain limitations. One of these limitations is the reliance on the VBN model to predict the intentions and behaviors of the study participants. Although the VBN model has been widely used in the field of agricultural technology adoption, it may not capture all the nuances of this particular population's attitudes and decision-making processes. Therefore, caution must be exercised when generalizing the findings to other contexts and populations. In future research, additional models and factors, such as the UTAUT model, should be considered to continue exploring and predicting the intention and adoption of hydroponic farming among youth groups.

Although all samples were drawn from multiple provinces and cities in China, the study sample size was limited to the population base in China. In addition, the 727 valid samples in this study did not adequately represent the entire population of unemployed youth in China. In addition, different provinces in China place different levels of importance on agricultural development, resulting in different knowledge and understanding of agricultural technology among youth groups in different regions, thus biases. Therefore, the results of the Ben study are limited to the current study sample and cannot be generalized to the entire Chinese population. Future research should focus on areas with better/worse agricultural development and improve the accuracy of the demographic attributes in the study results by narrowing the geographic scope to find appropriate methods and measures for agricultural development in different regions.

In addition, the measurement scales used in this study were adapted from existing literature, and most of the specific items were extracted from studies on green consumption (such as green foods and green products) and green tourism, rather than from studies oriented toward pro-environmental agricultural technologies (hydroponic farming techniques). Therefore, scale items in this study may have influenced the final results. Future studies need to optimize the measurement scales and thoroughly examine the linkages between related factors.

### Supplementary Information

Supplementary Information 1.

Supplementary Information 2.

Supplementary Information 3.

## Data Availability

The original contributions presented in the study are included in the article/Supplementary Material (Supporting Material S3. Dataset), further inquiries can be directed to the corresponding author/s.

## References

[1] Xu Z, Elomri A, Al-Ansari T, Kerbache L, El Mekkawy T (2022). Decisions on design and planning of solar-assisted hydroponic farms under various subsidy schemes. Renew. Sust. Energ. Rev..

[2] Singh R, Upadhyay SK, Aggarwal D, Sharma I, Prasad N (2019). A study on hydroponic farming system of wheat, spinach and sword lily for sustainable development of agriculture. Bio-Sci. Res. Bull..

[3] Khan S, Purohit A, Vadsaria N (2020). Hydroponics: Current and future state of the art in farming. J. Plant Nutr..

[4] Rahman MK, Bhuiyan MA, Zailani S (2021). Healthcare services: Patient satisfaction and loyalty lessons from Islamic Friendly Hospitals. Patient Prefer. Adherence..

[5] Rahman MK, Newaz MS, Hemmati M, Mallick SM (2021). Analyzing health-care service environment with malaysian general practice clinics. Health Educ..

[6] Sisodia GS, Alshamsi R, Sergi BS (2020). Business valuation strategy for new hydroponic farm development—A proposal towards sustainable agriculture development in United Arab Emirates. Br. Food J..

[7] Souza V (2023). Economic feasibility of adopting a hydroponics system on substrate in small rural properties. Clean Technol. Environ. Policy..

[8] Liu T, Yang M, Han Z, Ow DW (2016). Rooftop production of leafy vegetables can be profitable and less contaminated than farm-grown vegetables. Agron. Sustain. Dev..

[9] Lu Y, Zhang Y, Hong Y, He L, Chen Y (2022). Experiences and lessons from agri-food system transformation for sustainable food security: A review of China’s practices. Foods.

[10] Ahmed S, Hawarna S, Alqasmi I, Ashrafi DM, Rahman MK (2023). Mediating role of Lean management on the effects of workforce management and value-added time in private hospitals. Int. J. Lean Six Sigma..

[11] Stern PC (2000). New environmental theories: Toward a coherent theory of environmentally significant behavior. J. Soc. Issues..

[12] Steg L, Bolderdijk JW, Keizer K, Perlaviciute G (2014). An integrated framework for encouraging pro-environmental behaviour: The role of values, situational factors and goals. J. Environ. Psychol..

[13] Li L (2018). Exploring the residents’ intention to separate MSW in Beijing and understanding the reasons: An explanation by extended VBN theory. Sust. Cities Soc..

[14] Stern PC, Kalof L, Dietz T, Guagnano GA (1995). Values, beliefs, and proenvironmental action: Attitude formation toward emergent attitude objects. J. Appl. Soc. Psychol..

[15] López-Mosquera N, Sánchez M (2012). Theory of planned behavior and the value-belief-norm theory explaining willingness to pay for a suburban park. J. Environ. Manag..

[16] Choi H, Jang J, Kandampully J (2015). Application of the extended VBN theory to understand consumers’ decisions about Green Hotels. Int. J. Hosp. Manag..

[17] Ünal AB, Steg L, Granskaya J (2019). “To support or not to support, that is the question” testing the VBN theory in predicting support for car use reduction policies in Russia. Transp. Res. Part A Policy Pract..

[18] Wang J, Shen M, Chu M (2021). Why is green consumption easier said than done? exploring the green consumption attitude-intention gap in China with behavioral reasoning theory. Clean. Responsible Consum..

[19] Wang Y, Jiang Y, Geng B, Wu B, Liao L (2022). Determinants of returnees’ entrepreneurship in rural marginal China. J. Rural Stud..

[20] Chen Z, Sarkar A, Rahman A, Li X, Xia X (2022). Exploring the drivers of green agricultural development (GAD) in China: A spatial association network structure approaches. Land Pol..

[21] Jiang H-D, Yu R, Qian X-Y (2023). Socio-economic and energy-environmental impacts of technological change on China’s agricultural development under the Carbon Neutrality Strategy. Pet. Sci..

[22] Ajzen I (1991). The theory of planned behavior. Organ. Behav. Hum. Decis. Process..

[23] Nguyen T-M, Nham PT, Hoang V-N (2019). The theory of planned behavior and knowledge sharing. Vine J. Inf. Knowl. Manag. Syst..

[24] Kals E, Schumacher D, Montada L (1999). Emotional affinity toward nature as a motivational basis to protect nature. Environ. Behav..

[25] Xiao C, Dunlap RE, Hong D (2018). Ecological worldview as the central component of environmental concern: Clarifying the role of the NEP. Soc. Nat. Resour..

[26] Gkargkavouzi A, Halkos G, Matsiori S (2018). A multi-dimensional measure of environmental behavior: Exploring the predictive power of connectedness to nature, ecological worldview and environmental concern. Soc. Indic. Res..

[27] Kunchamboo V, Lee CK, Brace-Govan J (2021). Cultivating nature identity and ecological worldviews: A pathway to alter the prevailing dominant social paradigm. J. Macromarket..

[28] Lau RR, Hartman KA, Ware JE (1986). Health as a value: Methodological and theoretical considerations. Health Psychol..

[29] Lee J-S, Lee C-K, Choi Y (2010). Examining the role of emotional and functional values in festival evaluation. J. Travel Res..

[30] Li G, Yang L, Zhang B, Li X, Chen F (2021). How do environmental values impact green product purchase intention? the moderating role of Green Trust. Environ. Sci. Pollut. Res..

[31] Qasim H, Yan L, Guo R, Saeed A, Ashraf B (2019). The defining role of environmental self-identity among consumption values and behavioral intention to consume organic food. Int. J. Environ. Res. Public Health..

[32] Torres-Soto NY, Corral-Verdugo V, Corral-Frías NS (2022). The relationship between self-care, positive family environment, and human wellbeing. Wellbeing Space Soc..

[33] Ai Y (2022). Determinants of patients’ satisfaction and trust toward Healthcare Service Environment in general practice clinics. Front. Psychol..

[34] Mamun AA, Rahman MK, Munikrishnan UT, Permarupan PY (2021). Predicting the intention and purchase of health insurance among Malaysian working adults. SAGE Open.

[35] Han H, Hwang J (2016). What motivates delegates’ conservation behaviors while attending a convention?. J. Travel Tour. Mark..

[36] Donmez-Turan A, Kiliclar IE (2021). The analysis of pro-environmental behaviour based on ecological worldviews, environmental training/knowledge and goal frames. J. Clean Prod..

[37] Denley TJ (2020). Individuals’ intentions to engage in last chance tourism: Applying the value-belief-norm model. J. Sustain. Tour..

[38] Megeirhi HA, Woosnam KM, Ribeiro MA, Ramkissoon H, Denley TJ (2020). Employing a value-belief-norm framework to gauge carthage residents’ intentions to support sustainable cultural heritage tourism. J. Sustain. Tour..

[39] Van Riper CJ, Kyle GT (2014). Capturing multiple values of ecosystem services shaped by environmental worldviews: A spatial analysis. J. Environ. Manage..

[40] Han H (2015). Travelers’ pro-environmental behavior in a green lodging context: Converging value-belief-norm theory and the theory of planned behavior. Tourism Manage.

[41] Sharma R, Gupta A (2020). Pro-environmental behaviour among tourists visiting national parks: Application of value-belief-norm theory in an emerging economy context. Asia Pac. J. Tour. Res..

[42] De Groot JI, Steg L (2009). Morality and prosocial behavior: The role of awareness, responsibility, and norms in the norm activation model. J. Social Psychol..

[43] Wu L (2018). The relationships between environmental sensitivity, ecological worldview, personal norms and Pro-environmental behaviors in Chinese children: Testing the value–belief–norm model with environmental sensitivity as an emotional basis. Psych. J..

[44] Kwame Yeboah F, Kaplowitz M (2016). Explaining energy conservation and environmental citizenship behaviors using the value-belief-norm framework. Hum. Ecol. Rev..

[45] Riepe C (2021). Values, beliefs, norms, and conservation-oriented behaviors toward native fish biodiversity in rivers: Evidence from four European countries. Soc. Nat. Resour..

[46] Wu L, Zhu Y (2021). How love of nature promotes green consumer behaviors: The mediating role of biospheric values, ecological worldview, and personal norms. Psych J..

[47] Han H (2020). Theory of green purchase behavior (TGPB): A new theory for sustainable consumption of Green Hotel and Green Restaurant Products. Bus. Strateg. Environ..

[48] Chen Y (2020). An investigation of the influencing factors of Chinese WeChat users’ environmental information-sharing behavior based on an integrated model of UGT, Nam, and TPB. Sustainability.

[49] He X, Zhan W (2018). How to activate moral norm to adopt electric vehicles in China? an empirical study based on extended norm activation theory. J. Clean Prod..

[50] Kim SH, Seock Y-K (2019). The roles of values and social norm on personal norms and pro-environmentally friendly apparel product purchasing behavior: The mediating role of personal norms. J. Retail. Consum. Serv..

[51] Koklic MK, Golob U, Podnar K, Zabkar V (2019). The interplay of past consumption, attitudes and personal norms in organic food buying. Appetite..

[52] Jeong J, Kim Y, Roh T (2021). Do consumers care about aesthetics and compatibility? the intention to use wearable devices in health care. SAGE Open..

[53] Senali MG (2022). Determinants of intention to use e-wallet: Personal innovativeness and propensity to trust as moderators. Int. J. Hum.-Comput. Interact..

[54] Al-Bashayreh M, Almajali D, Altamimi A, Masa’deh R, Al-Okaily M (2022). An empirical investigation of reasons influencing student acceptance and rejection of mobile learning apps usage. Sustainability.

[55] Shi S, Wang Y, Chen X, Zhang Q (2020). Conceptualization of omnichannel customer experience and its impact on shopping intention: A mixed-method approach. Int. J. Inf. Manage..

[56] Yang Q (2022). Predicting the mass adoption of eDoctor apps during COVID-19 in China using hybrid sem-neural network analysis. Front. Public Health.

[57] Kaiser FG, Gutscher H (2003). The proposition of a general version of the theory of planned behavior: Predicting ecological behavior1. J. Appl. Soc. Psychol..

[58] Liu P, Teng M, Han C (2020). How does environmental knowledge translate into pro-environmental behaviors?: The mediating role of environmental attitudes and behavioral intentions. Sci. Total Environ..

[59] Chwialkowska A, Bhatti WA, Glowik M (2020). The influence of cultural values on pro-environmental behavior. J. Clean Prod..

[60] Laureti T, Benedetti I (2018). Exploring pro-environmental food purchasing behaviour: An empirical analysis of Italian consumers. J. Clean Prod..

[61] Sweeney JC, Soutar GN (2001). Consumer perceived value: The development of a multiple item scale. J. Retail..

[62] Al Mamun A, Naznen F, Jingzu G, Yang Q (2023). Predicting the intention and adoption of hydroponic farming among Chinese urbanites. Heliyon.

[63] Maichum K, Parichatnon S, Peng K-C (2016). Application of the extended theory of planned behavior model to investigate purchase intention of green products among Thai consumers. Sustainability.

[64] Kim H, Kim J, Oh KW, Jung HJ (2016). Adoption of eco-friendly faux leather. Cloth. Text. Res. J..

[65] Kock N (2015). Common method bias in PLS-SEM. Int. J. e-Collab..

[66] Podsakoff PM, MacKenzie SB, Podsakoff NP (2012). Sources of method bias in social science research and recommendations on how to control it. Annu. Rev. Psychol..

[67] Hair JF (2021). Partial least squares structural equation modeling (PLS-SEM) using r: A workbook.

[68] Becker J-M, Cheah J-H, Gholamzade R, Ringle CM, Sarstedt M (2022). PLS-SEM’s most wanted guidance. Int. J. Contemp. Hosp. Manag..

[69] Hair JF, Risher JJ, Sarstedt M, Ringle CM (2019). When to use and how to report the results of PLS-SEM. Eur. Bus. Rev..

[70] Avkiran NK (2017). An in-depth discussion and illustration of partial least squares structural equation modeling in health care. Health Care Manag. Sci..

[71] Sarstedt M (2022). Progress in partial least squares structural equation modeling use in marketing research in the last decade. Psychol. Mark..

[72] Zhang L, Ruiz-Menjivar J, Luo B, Liang Z, Swisher ME (2020). Predicting climate change mitigation and adaptation behaviors in agricultural production: A comparison of the theory of planned behavior and the value-belief-norm theory. J Environ Psychol..

[73] Cao H, Li F, Zhao K, Qian C, Xiang T (2022). From value perception to behavioural intention: Study of Chinese smallholders’ pro-environmental agricultural practices. J. Environ. Manage..

