# Supplementary material for: Environmental and health values, beliefs, norms and compatibility on intention to adopt hydroponic farming among unemployed youth
Source: Sci Rep. 2024 Jan 18;14:1592. doi: 10.1038/s41598-024-52064-w (PMC10796339; doi:10.1038/s41598-024-52064-w)
Supplement: Supplementary file 1 — Supplementary Information 1. [file 41598_2024_52064_MOESM1_ESM.docx]

***Supporting Material S1****. Survey Instrument*

| Code | Items |
| --- | --- |
| Environmental Values | |
| EN1 | Humans need to understand how nature works and adapt to nature. |
| EN2 | We should live in harmony with nature. |
| EN3 | Humans are only part of nature. |
| EN4 | Unless each of us recognizes the need to protect the environment, our future generations will bear the consequences. |
| Emotional Values | |
| EM1 | It was enjoyable to work in the agricultural farms. |
| EM2 | I felt better after visiting the agricultural farms. |
| EM3 | Overall, it was beneficial and worthwhile to see the agricultural farms. |
| EM4 | Working in agricultural farms is something I'd like to do. |
| EM5 | It would make me happy to work on an agricultural farm. |
| Health Values | |
| HV1 | If I don’t have my health, I don’t have anything |
| HV2 | There is nothing I care more about than my health |
| HV3 | Good health is most important for happy life |
| HV4 | Nothing is more important than good health |
| Ecological Worldview | |
| EW1 | When humans interfere with nature, the consequences can be disastrous |
| EW2 | Plants and animals have as much right to live as humans |
| EW3 | Conventional food production is seriously abusing the environment |
| EW4 | The balance of nature is very delicate and easily upset |
| EW5 | Human is responsible for the long-life of nature sustainability |
| Awareness of Consequences | |
| AC1 | Global warming is a problem for society |
| AC2 | Energy savings help reduce global warming |
| AC3 | Environmental quality will improve if we organic food production method |
| AC4 | Protection of the environment benefits us all |
| AC5 | Organic food production method is beneficial for our health |
| AC6 | Organic food production method improves our quality of life |
| Ascription of Responsibility | |
| AR1 | We are jointly responsible for global warming |
| AR2 | We are jointly responsible for the environmental problems caused by the fossil fuel industry |
| AR3 | We are jointly responsible for the environmental problems caused by convention food production practices |
| AR4 | We are jointly responsible for the environmental deterioration caused by convention food production practices |
| AR5 | We are jointly responsible for the ecological deterioration caused by convention food production practices |
| Personal Norms | |
| PN1 | I feel morally obliged to adopt organic food production methods |
| PN2 | People like me should do everything they can to save the environment |
| PN3 | I feel obliged to bear the environment and nature in mind in my consumption behaviours |
| PN4 | I feel morally obliged to use organic products, regardless of what others do |
| PN5 | I feel personally obliged to save as much energy as possible |
| Intention towards Hydroponic Farming | |
| IT1 | The hydroponic farming method is what I intend to use. |
| IT2 | In the foreseeable future, I am willing to employ hydroponic farming. |
| IT3 | In the near future, I will continue to use hydroponic gardening. |
| IT4 | I will tell people about hydroponic farming. |
| IT5 | Because I recognize the advantages of hydroponic farming, I aim to use it. |
| Hydroponics Compatibility | |
| HC1 | I have all of the resources I need to apply hydroponic farming techniques. |
| HC2 | I have the information required to apply hydroponic farming techniques. |
| HC3 | My hydroponic system is compatible with my urban garden. |
| HC4 | When I'm having trouble using the hydroponic system on my farm, I can seek assistance from others. |
| HC5 | I believe that I have complete control over my hydroponic system. |
| Adoption of Hydroponic Farming | |
| AD1 | Because I want a high-quality produce, I employ hydroponic farming. |
| AD2 | For maximum efficiency, I employ a hydroponic farming system. |
| AD3 | To reduce environmental impact, I employ hydroponic farming. |
| AD4 | Because I am concerned about my health and the environment, I employ hydroponic farming. |
