# Supplementary material for: Environmental and health values, beliefs, norms and compatibility on intention to adopt hydroponic farming among unemployed youth
Source: Sci Rep. 2024 Jan 18;14:1592. doi: 10.1038/s41598-024-52064-w (PMC10796339; doi:10.1038/s41598-024-52064-w)
Supplement: Supplementary file 2 — Supplementary Information 2. [file 41598_2024_52064_MOESM2_ESM.docx]

**Supporting Material S2.** Discriminant Validity

|  | | EN | | EM | | HV | | EW | | AC | | AR | | PN | | IT | | HC | | AD | |
| --- | --- | --- | --- | --- | --- | --- | --- | --- | --- | --- | --- | --- | --- | --- | --- | --- | --- | --- | --- | --- | --- |
| Heterotrait-monotrait ratio (HTMT) - Matrix | | | | | | | | | | | | | |  | |  | |  | |  | |
| EN | |  | |  | |  | |  | |  | |  | |  | |  | |  | |  | |
| EM | | 0.447 | |  | |  | |  | |  | |  | |  | |  | |  | |  | |
| HV | | 0.352 | | 0.322 | |  | |  | |  | |  | |  | |  | |  | |  | |
| EW | | 0.411 | | 0.485 | | 0.468 | |  | |  | |  | |  | |  | |  | |  | |
| AC | | 0.366 | | 0.476 | | 0.371 | | 0.387 | |  | |  | |  | |  | |  | |  | |
| AR | | 0.421 | | 0.423 | | 0.413 | | 0.437 | | 0.351 | |  | |  | |  | |  | |  | |
| PN | | 0.391 | | 0.468 | | 0.426 | | 0.449 | | 0.398 | | 0.418 | |  | |  | |  | |  | |
| IT | | 0.341 | | 0.412 | | 0.340 | | 0.314 | | 0.344 | | 0.351 | | 0.366 | |  | |  | |  | |
| HC | | 0.260 | | 0.262 | | 0.273 | | 0.238 | | 0.227 | | 0.246 | | 0.260 | | 0.284 | |  | |  | |
| AD | | 0.374 | | 0.367 | | 0.391 | | 0.393 | | 0.399 | | 0.348 | | 0.398 | | 0.505 | | 0.456 | |  | |
| Fornell-Larcker criterion | | | | | | | | | | | |  | |  | |  | |  | |  | |
| EN | | 0.864 | |  | |  | |  | |  | |  | |  | |  | |  | |  | |
| EM | | 0.396 | | 0.825 | |  | |  | |  | |  | |  | |  | |  | |  | |
| HV | | 0.309 | | 0.287 | | 0.847 | |  | |  | |  | |  | |  | |  | |  | |
| EW | | 0.361 | | 0.430 | | 0.408 | | 0.809 | |  | |  | |  | |  | |  | |  | |
| AC | | 0.321 | | 0.415 | | 0.322 | | 0.336 | | 0.760 | |  | |  | |  | |  | |  | |
| AR | | 0.370 | | 0.373 | | 0.361 | | 0.381 | | 0.309 | | 0.812 | |  | |  | |  | |  | |
| PN | | 0.342 | | 0.411 | | 0.369 | | 0.389 | | 0.341 | | 0.364 | | 0.799 | |  | |  | |  | |
| IT | | 0.291 | | 0.347 | | 0.284 | | 0.262 | | 0.289 | | 0.294 | | 0.304 | | 0.748 | |  | |  | |
| HC | | 0.227 | | 0.225 | | 0.232 | | 0.202 | | 0.194 | | 0.206 | | 0.221 | | 0.234 | | 0.781 | |  | |
| AD | | 0.336 | | 0.328 | | 0.344 | | 0.347 | | 0.353 | | 0.308 | | 0.348 | | 0.429 | | 0.395 | | 0.867 | |
| Loading and Cross Loadings | | | | | | | | | | | | | | | | | | | | |  |
| EN1 | 0.897 | | 0.374 | | 0.281 | | 0.347 | | 0.310 | | 0.317 | | 0.315 | | 0.281 | | 0.247 | | 0.350 | |  |
| EN2 | 0.841 | | 0.323 | | 0.254 | | 0.282 | | 0.239 | | 0.295 | | 0.276 | | 0.195 | | 0.133 | | 0.225 | |  |
| EN3 | 0.851 | | 0.311 | | 0.278 | | 0.304 | | 0.277 | | 0.323 | | 0.276 | | 0.231 | | 0.197 | | 0.296 | |  |
| EN4 | 0.868 | | 0.356 | | 0.253 | | 0.310 | | 0.280 | | 0.344 | | 0.313 | | 0.292 | | 0.197 | | 0.279 | |  |
| EM1 | 0.320 | | 0.844 | | 0.275 | | 0.379 | | 0.338 | | 0.317 | | 0.315 | | 0.299 | | 0.208 | | 0.286 | |  |
| EM2 | 0.340 | | 0.853 | | 0.290 | | 0.377 | | 0.382 | | 0.313 | | 0.360 | | 0.340 | | 0.201 | | 0.323 | |  |
| EM3 | 0.348 | | 0.813 | | 0.189 | | 0.316 | | 0.305 | | 0.308 | | 0.316 | | 0.266 | | 0.150 | | 0.243 | |  |
| EM4 | 0.322 | | 0.840 | | 0.216 | | 0.394 | | 0.321 | | 0.337 | | 0.392 | | 0.263 | | 0.165 | | 0.268 | |  |
| EM5 | 0.308 | | 0.772 | | 0.201 | | 0.286 | | 0.373 | | 0.256 | | 0.300 | | 0.261 | | 0.206 | | 0.223 | |  |
| HV1 | 0.262 | | 0.308 | | 0.860 | | 0.364 | | 0.288 | | 0.342 | | 0.356 | | 0.246 | | 0.162 | | 0.279 | |  |
| HV2 | 0.227 | | 0.179 | | 0.843 | | 0.335 | | 0.281 | | 0.265 | | 0.292 | | 0.235 | | 0.202 | | 0.281 | |  |
| HV3 | 0.279 | | 0.217 | | 0.821 | | 0.318 | | 0.256 | | 0.299 | | 0.271 | | 0.257 | | 0.223 | | 0.303 | |  |
| HV4 | 0.278 | | 0.260 | | 0.864 | | 0.360 | | 0.264 | | 0.314 | | 0.326 | | 0.227 | | 0.202 | | 0.302 | |  |
| EW1 | 0.298 | | 0.311 | | 0.351 | | 0.780 | | 0.228 | | 0.296 | | 0.294 | | 0.252 | | 0.137 | | 0.264 | |  |
| EW2 | 0.263 | | 0.354 | | 0.347 | | 0.827 | | 0.310 | | 0.319 | | 0.358 | | 0.217 | | 0.139 | | 0.287 | |  |
| EW3 | 0.296 | | 0.347 | | 0.344 | | 0.828 | | 0.270 | | 0.279 | | 0.335 | | 0.212 | | 0.159 | | 0.293 | |  |
| EW4 | 0.325 | | 0.362 | | 0.290 | | 0.796 | | 0.259 | | 0.323 | | 0.269 | | 0.197 | | 0.216 | | 0.248 | |  |
| EW5 | 0.283 | | 0.363 | | 0.314 | | 0.811 | | 0.287 | | 0.326 | | 0.312 | | 0.185 | | 0.170 | | 0.308 | |  |
| AC1 | 0.243 | | 0.297 | | 0.243 | | 0.230 | | 0.799 | | 0.238 | | 0.276 | | 0.209 | | 0.148 | | 0.282 | |  |
| AC2 | 0.209 | | 0.328 | | 0.247 | | 0.255 | | 0.774 | | 0.170 | | 0.258 | | 0.216 | | 0.158 | | 0.268 | |  |
| AC3 | 0.214 | | 0.280 | | 0.264 | | 0.234 | | 0.723 | | 0.231 | | 0.260 | | 0.193 | | 0.121 | | 0.209 | |  |
| AC4 | 0.260 | | 0.375 | | 0.270 | | 0.306 | | 0.783 | | 0.298 | | 0.277 | | 0.258 | | 0.186 | | 0.330 | |  |
| AC5 | 0.275 | | 0.329 | | 0.250 | | 0.253 | | 0.755 | | 0.263 | | 0.264 | | 0.262 | | 0.145 | | 0.319 | |  |
| AC6 | 0.257 | | 0.268 | | 0.183 | | 0.245 | | 0.723 | | 0.188 | | 0.215 | | 0.164 | | 0.118 | | 0.179 | |  |
| AR1 | 0.244 | | 0.289 | | 0.306 | | 0.285 | | 0.237 | | 0.799 | | 0.282 | | 0.267 | | 0.201 | | 0.288 | |  |
| AR2 | 0.323 | | 0.298 | | 0.296 | | 0.361 | | 0.280 | | 0.859 | | 0.342 | | 0.239 | | 0.145 | | 0.262 | |  |
| AR3 | 0.307 | | 0.282 | | 0.258 | | 0.284 | | 0.206 | | 0.756 | | 0.241 | | 0.212 | | 0.216 | | 0.214 | |  |
| AR4 | 0.303 | | 0.322 | | 0.302 | | 0.292 | | 0.277 | | 0.832 | | 0.294 | | 0.256 | | 0.146 | | 0.238 | |  |
| AR5 | 0.324 | | 0.324 | | 0.301 | | 0.320 | | 0.248 | | 0.812 | | 0.308 | | 0.220 | | 0.147 | | 0.248 | |  |
| PN1 | 0.305 | | 0.349 | | 0.310 | | 0.315 | | 0.272 | | 0.308 | | 0.838 | | 0.281 | | 0.184 | | 0.275 | |  |
| PN2 | 0.306 | | 0.375 | | 0.284 | | 0.340 | | 0.257 | | 0.321 | | 0.817 | | 0.234 | | 0.191 | | 0.279 | |  |
| PN3 | 0.224 | | 0.290 | | 0.304 | | 0.297 | | 0.289 | | 0.267 | | 0.779 | | 0.237 | | 0.147 | | 0.284 | |  |
| PN4 | 0.253 | | 0.332 | | 0.301 | | 0.292 | | 0.253 | | 0.286 | | 0.760 | | 0.237 | | 0.160 | | 0.252 | |  |
| PN5 | 0.275 | | 0.291 | | 0.276 | | 0.309 | | 0.293 | | 0.271 | | 0.797 | | 0.222 | | 0.200 | | 0.300 | |  |
| IT1 | 0.300 | | 0.318 | | 0.303 | | 0.266 | | 0.278 | | 0.275 | | 0.294 | | 0.758 | | 0.195 | | 0.297 | |  |
| IT2 | 0.260 | | 0.338 | | 0.274 | | 0.247 | | 0.243 | | 0.292 | | 0.274 | | 0.712 | | 0.126 | | 0.276 | |  |
| IT3 | 0.125 | | 0.172 | | 0.132 | | 0.113 | | 0.145 | | 0.147 | | 0.169 | | 0.728 | | 0.189 | | 0.315 | |  |
| IT4 | 0.197 | | 0.213 | | 0.193 | | 0.184 | | 0.202 | | 0.215 | | 0.209 | | 0.783 | | 0.184 | | 0.368 | |  |
| IT5 | 0.201 | | 0.259 | | 0.157 | | 0.165 | | 0.209 | | 0.169 | | 0.189 | | 0.755 | | 0.177 | | 0.342 | |  |
| HC1 | 0.213 | | 0.177 | | 0.153 | | 0.146 | | 0.089 | | 0.167 | | 0.136 | | 0.180 | | 0.787 | | 0.309 | |  |
| HC2 | 0.218 | | 0.194 | | 0.180 | | 0.176 | | 0.156 | | 0.211 | | 0.185 | | 0.186 | | 0.770 | | 0.317 | |  |
| HC3 | 0.180 | | 0.193 | | 0.189 | | 0.168 | | 0.149 | | 0.165 | | 0.183 | | 0.160 | | 0.783 | | 0.309 | |  |
| HC4 | 0.162 | | 0.179 | | 0.211 | | 0.173 | | 0.194 | | 0.172 | | 0.204 | | 0.194 | | 0.789 | | 0.293 | |  |
| HC5 | 0.113 | | 0.137 | | 0.172 | | 0.126 | | 0.170 | | 0.090 | | 0.155 | | 0.192 | | 0.774 | | 0.315 | |  |
| AD1 | 0.290 | | 0.290 | | 0.301 | | 0.321 | | 0.313 | | 0.288 | | 0.301 | | 0.389 | | 0.357 | | 0.862 | |  |
| AD2 | 0.262 | | 0.268 | | 0.282 | | 0.292 | | 0.279 | | 0.249 | | 0.290 | | 0.364 | | 0.325 | | 0.865 | |  |
| AD3 | 0.302 | | 0.273 | | 0.284 | | 0.280 | | 0.316 | | 0.226 | | 0.287 | | 0.351 | | 0.335 | | 0.862 | |  |
| AD4 | 0.310 | | 0.306 | | 0.324 | | 0.308 | | 0.316 | | 0.301 | | 0.328 | | 0.381 | | 0.354 | | 0.880 | |  |

**Note:** EN: Environmental Values; EM: Emotional Values; HV: Health Values; EW: Ecological Worldview; AC: Awareness of Consequences; AR: Ascription of Responsibility; PN: Personal Norms; IT: Intention towards Hydroponic Farming; HC: Hydroponics Compatibility; AD: Adoption of Hydroponic Farming.

**Source:** Author’s data analysis.
